# Nanomedicine Fight against Antibacterial Resistance: An Overview of the Recent Pharmaceutical Innovations

**DOI:** 10.3390/pharmaceutics12020142

**Published:** 2020-02-08

**Authors:** Nermin E. Eleraky, Ayat Allam, Sahar B. Hassan, Mahmoud M. Omar

**Affiliations:** 1 Department of Pharmaceutics, Faculty of Pharmacy, Assiut University, Assiut 71526, Egypt; nermineleraky@pharm.aun.edu.eg (N.E.E.); allamayat@yahoo.com (A.A.); 2 Assiut International Center of Nanomedicine, Al-Rajhy Liver Hospital, Assiut University, Assiut 71515, Egypt; 3 Department of Clinical pharmacy, Faculty of Pharmacy, Assiut University, Assiut 71526, Egypt; saharbadr164@yahoo.com; 4 Department of Pharmaceutics and Industrial Pharmacy, Deraya University, Minia 61768, Egypt; 5 Department of Pharmaceutics and Clinical Pharmacy, Faculty of Pharmacy Sohag University, Sohag 82524, Egypt

**Keywords:** nanomedicine, antibacterial resistance, inhibition of antibacterial resistance, anti-biofilm mechanisms, organic nanosystems, inorganic nanosystems

## Abstract

Based on the recent reports of World Health Organization, increased antibiotic resistance prevalence among bacteria represents the greatest challenge to human health. In addition, the poor solubility, stability, and side effects that lead to inefficiency of the current antibacterial therapy prompted the researchers to explore new innovative strategies to overcome such resilient microbes. Hence, novel antibiotic delivery systems are in high demand. Nanotechnology has attracted considerable interest due to their favored physicochemical properties, drug targeting efficiency, enhanced uptake, and biodistribution. The present review focuses on the recent applications of organic (liposomes, lipid-based nanoparticles, polymeric micelles, and polymeric nanoparticles), and inorganic (silver, silica, magnetic, zinc oxide (ZnO), cobalt, selenium, and cadmium) nanosystems in the domain of antibacterial delivery. We provide a concise description of the characteristics of each system that render it suitable as an antibacterial delivery agent. We also highlight the recent promising innovations used to overcome antibacterial resistance, including the use of lipid polymer nanoparticles, nonlamellar liquid crystalline nanoparticles, anti-microbial oligonucleotides, smart responsive materials, cationic peptides, and natural compounds. We further discuss the applications of antimicrobial photodynamic therapy, combination drug therapy, nano antibiotic strategy, and phage therapy, and their impact on evading antibacterial resistance. Finally, we report on the formulations that made their way towards clinical application.

## 1. Introduction

The resistance to antibiotics is defined as the ability of bacteria causing disease to resist the therapeutic effects of antibacterial drugs. The danger of antibiotic resistance comes from; it resulted in enormous human and economic losses. About 700,000 people have died each year worldwide thanks to the inappropriate antibiotic usage that develops resistance to conventional therapy [1]. For instance, *Staphylococcus aureus* (MRSA) that resist methicillin was reported to cause almost 120,000 blood-borne infections and 20,000 related deaths in the United States in 2017 [2]. Moreover, carbapenem-resistant Enterobacteriaceae (CRE) has been regarded as a public health threat that requires prompt and invasive actions [3].

Antibiotic-resistant infections were reported to give rise to losses estimated at $55–70 billion annually in USA. In Europe, the losses surpassed €1.5 billion annually [4,5]. The excessive and improper consumption of antibacterials resulted in the emergence of more aggressive strains that do not respond to standard treatments [6]. In addition, there are many concerns related to conventional antibacterial drug usage; such as low water solubility, diminished stability, minimum oral bioavailability, drug targeting complexity, and depressed patient compliance as a result of frequent drug administration and variable toxicity [7]. The disastrous human and economic cost of antibiotic resistance renders the development of newalternative strategies more urgent in order to confront this massive challenge.

To highlight the application of nanosystems as antibacterial delivery agents, it is worth identifying the mechanism by which bacteria form colonies that escape conventional antibiotic therapies. Two forms of bacterial growth exist; the first form is the planktonic growth, which is characterized by a free-swimming unicellular phase existence that is not attached to a surface; while the second form is the biofilm growth phase, which is described as a multicellular sessile state that forms communities [8]. Biofilm represents an evolved system that permits bacteria to be alive in hostile environments, forming permanent colonies, with high ability to dissociate and form new colonies [9,10].

Biofilm bacterial growth is composed of a dense and hydrated group of bacteria attached to each other and to a surface where they are surrounded by an external matrix composed of exo polysaccharide, amino acids, and extracellular deoxyribonucleic acid (DNA) [11]. It is considered to be 1000 times more resilient to conventional antibiotic treatments relative to planktonic bacterial growth [12]. Biofilm is associated with many diseases such as lung, colon, urethra, eye, and ear infections, in addition to infective endocarditis, gum-related infections, and wound-related infections [13]. Biofilm bacteria are liable to cell-density-dependent regulation from its extracellular polymeric substances (EPS) matrix; consequently, they are released into the external environment as free-floating bacteria. Moreover, activation of the normal nonpathogenic commensal bacteria of the human body into virulent forms is facilitated by both biofilms and immune responses of host [14]. Increased genetic mutations rates within biofilms assist the development of survival mechanisms. For example, up-regulation of proteins and expression of particular efflux pumps might diffuse across the biofilm. Moreover, elevated expression of toxin–antitoxin modules stops key cell functions such as translation [15,16]. Due to the diversity and anonymous biofilm-resistant mechanisms, innovative nanosystems should be developed to stop the spread of resistant bacterial infections.

The present review will discuss role of nanosystems in overcoming the bacterial resistance and will outline the various mechanisms of nanosystems as antibacterial drug delivery agents. These nanosystems are classified into two categories; the first one is organic nanosystems such as liposomes, lipid-based nanoparticles, polymeric micelles, and polymeric nanoparticles, and the second one is inorganic nanosystems such as silver, silica, magnetic, zinc oxide (ZnO), cobalt, selenium, and cadmium nanoparticles. Clinical trials and challenges in the clinical translation of nanomedicines will also be discussed.

## 2. Nanosystems’ Role in Overcoming Antibiotic Resistance

The emergence of aggressive bacteria together with the limited production of new antibacterial drugs has resulted in inefficiency of current antibiotic therapy with relevant risks on human health. The availability of new antibacterial agents appeared to be a very complex process in view of the capability to produce new effective and safe drugs, in addition to the high production costs and the time required for approval of new drugs that takes about 10–15 years [7]. In 2016, many antibiotics were clinically tested for the market in the United States of America [1]. Sadly, however, in the last decades, linezolid was the only approved antibiotic together with the recently discovered teixobactin [17].

Based on the aforementioned facts, the current researches are aimed towards the discovery of novel techniques to overcome these relevant challenges and, hence, the efficiency of conventional antibacterial drugs will be improved. Nanomedicine plays a vital role in enhancing the effectiveness of existent therapeutics, by enhancing the physicochemical properties and stability of antibiotics, offering a chance of biofilm internalization, prolongation of antibiotic release, in addition to the capability of targeted delivery to the site of infection and improved systemic circulation with a consequent reduction of the related side effects compared to the corresponding free drugs [18].

### 2.1. Mechanism of Nanosystems as Antibacterial Drug Delivery Agents

The physicochemical properties of nanosystems, particle size, surface charge, and solubility, are the key factors that control vital processes for example intracellular uptake, biodistribution, or clearance. Nanometer-sized particles enable better drug loading efficiency of both hydrophilic and lipophilic antibiotics and hence enhanced antibacterial effect [19]. In addition to a more expected cellular internalization of the antibiotics loaded nanosystems was achieved by passing the reticulo-endothelial system [20]. Surface charge and the zeta (ζ)-potential of nanosystems drives interactions with proteins, tissues, or with various components of the tissue, thus affecting cellular biodistribution and uptake. Host cells such as macrophages with anionic nature thus attract positively charged nanosystems compared to uncharged and negatively charged ones [21].

Hydrophobicity of nanosystems plays a great function in targeting of the drug delivery related to interactions with the phospholipid layer of the bacterial membrane [18]. On the contrary, hydrophilic nanosystems interact less with opsonins thus, having longer blood circulation compared with hydrophobic nanosystems [22]. Thus, the enhanced actions of nanosystems as antibacterial drug delivery systems arise from various mechanisms, including their ability to optimize the physicochemical characteristics of entrapped antibacterial drugs, their favored accumulation near the cytoplasm, their electrostatic interactions with bacterial membrane, the high oxidizing power and production of reactive oxygen species, the prevention of unwanted interactions and protection of antibacterials against degradation and the better clinical use of antibacterials through more patient acceptable routes [23].

Interestingly, it was found that nano-sized systems not only improve the therapeutic activity of antibacterial agents but also restrain the stimulation of resistance by overcoming bacteria developed resistance strategies that involve drug decomposition by β-lactamase, efflux pumps, or thickening of bacterial cell walls [24].

### 2.2. Classification of Nanosystems

**Figure 1:**
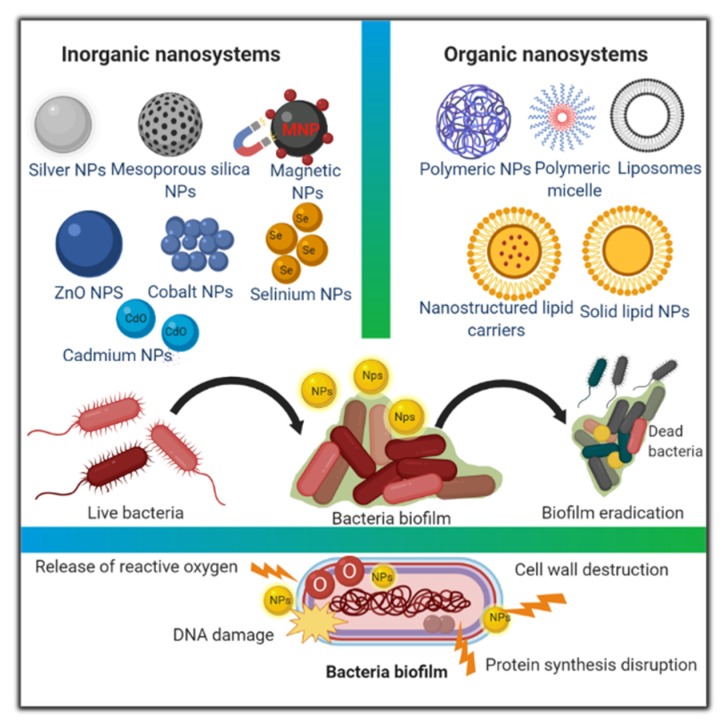
Graphical outline of various classes of nanosystems with illustration of their possible anti-biofilm mechanisms.

Nanosystems can be categorized based on their matrix properties and the material constituting them into inorganic and organic nanosystems [25], (Figure 1).

Inorganic nanosystems represent a class of nanosystems that originates from inorganic oxides. Their synthesis technique depends on chemical reduction of metallic salts with a reductant. The reaction environmental parameters, for example temperature and pH, play a major function in determining the specificities of these materials, consequently affecting their loading capacity, the in vitro drug release kinetics, aggregation, and hence their antibacterial effect [26].

Furthermore, organic nanosystems such as liposomes, lipid-based nanoparticles, polymeric micelles and polymeric nanoparticles have preferable biodegradability and biocompatibility features, making them suitable candidates for clinical use [27]. Herein, we will report on the recent updates of organic and inorganic nanosystems as antibacterial drug delivery systems with a brief description of each system. Then, we will briefly discuss the recent trends used to overcome antibacterial resistance.

#### 2.2.1. Organic Nanosystems

##### Liposomes

• Composition and characteristics of liposomes

Liposomes are considered the most extensively evaluated antimicrobial drug delivery nanosystems. They are characterized by spherical structures made up of phospholipid bilayer(s) surrounding an inner aqueous space, ranging in size from 0.02 to 10 µm [28,29,30]. The efficacy of antibacterial-loaded liposomes in biofilm eradication relies on the physicochemical properties of liposomes that control their stability and in vivo interactions [31]. Moreover, liposomes are regarded as inclusive carriers for both hydrophilic and hydrophobic therapeutics. Large unilamellar vesicles including a large volume of aqueous phase are the best carrier for hydrosoluble agents, while hydrophobic compounds can be enclosed in the lipid bilayer of multilamellar or small unilamellar vesicles [32,33].

For antibiotic delivery small unilamellar vesicles of ≃100 nm displayed high capability in the eradication of bacterial strains [34]. Liposomes proved to be useful for the management of topical [35], vaginal [36], pulmonary [37], and ocular [38] bacterial infections.

• Advantages of antibiotics-loaded liposomes as drug delivery agents:

1- Better protection and enhanced antibiotics biodistribution.

Liposomes improved antibiotics pharmacokinetics and pharmacodynamics in a way where inclusion within the liposomal vesicles controls and sustains drug release, maintaining proper antibiotic level for a long enough time, on the contrary to free antibiotics administration that requires several doses per day thus minimizing patient adherence to therapy [39]. In addition, encapsulation of antibiotics within liposomal vesicles safeguards antibiotics against the degradative effect of the defense mechanisms of the body, thus preserving their therapeutic response [40].

In an attempt to enhance the stabilization of orally administered peptide antibiotics, vancomycin was encapsulated within liposomes containing specific tetra ether lipid. The results of in vivo study on Wistar rats expressed a strong enhancement in the oral bioavailability of vancomycin using the liposomal formulation (4.82 ± 0.56%), where the given oral dose of vancomycin reached the blood after one hour, which is considered a very good achievement for the oral administration of peptide antibiotics [41]. Further, administrations of either dicloxacillin-loaded liposomes or dicloxacillin-loaded chitosan-coated liposomes were evaluated against MRSA infections. A significantly wider zone of inhibition of dicloxacillin-loaded liposomes compared to free drug and drug-loaded chitosan-coated liposomes (55.0 ± 1.70, 34.3 ± 0.5, 33.0 ± 0.89 mm, respectively) confirmed the better antibacterial activity of small-sized liposomes as well as better drug biodistribution. Nevertheless, testing formulations in vivo on an MRSA infected animal model is recommended [42].

2- Selective biofilm targeting affinity.

The surface structure of liposomes specifies the type of interaction with the target bacterial biofilm. For nonspecific interactions, the charge of the liposome membrane plays a vital role. Consequently, the liposomes with positive charge showed the strongest interactions with the negatively charged bacterial biofilms. However, for a specific interaction with the target, liposomes are usually equipped with either proteins, antibodies, specific oligosaccharide chains, or immunoglobulin fragments that express an affinity to certain receptors located on the target biofilm, in addition to the possibility of formulating pH-sensitive or thermo-sensitive liposomes vesicles [31].

To improve the gastrointestinal targeting affinity, Wenxi Wang et al. [43] designed S-layer proteins coated positively charged liposomes. S-layer proteins are crystalline arrays of self-assembled protein located on the surface of bacterial cell, that have the ability to bond to cationic liposomes through their carboxyl groups, and then self-reassemble as a functional coat of liposomes. The authors revealed that coating liposomes with S-layer proteins results in significant improvement of the gastrointestinal adhesion property. Wheat germ agglutinin-conjugated liposomes with surface grafted cyclodextrin were developed to overcome oral infections. Two physicochemical variable drugs (ciprofloxacin and betamethasone) were successfully encapsulated and showed a prolonged co-drug release in saliva over a period of 24 h and a significant increase in oral cell survival against Aggregatibacter actinomycetemcomitans biofilm combined with reduced inflammation [44].

Moayad Alhariri et al. [45] tested the targeting efficiency of neutral and negatively charged gentamicin-loaded liposomes towards *P. aeruginosa* (*Pseudomonas aeruginosa*) and *K. oxytoca* (*Klebsiella oxytoca*) pathogenic strains. Surprisingly, it was found that anionic liposomes improved drug encapsulation and enhanced the targeting affinity of gentamicin to bacterial biofilm better than either neutral antibiotic-loaded liposomes or free gentamicin. This could be better interpreted based on the increased encapsulation efficiency of the positively charged antibiotic (gentamicin) within negatively charged liposomes based on the electrostatic interaction, followed by improved delivery of antibiotic-loaded negatively charged liposomes through a fusion mechanism that allows the direct injection of liposome-entrapped antibiotic into the cytoplasm of bacteria despite the repulsive forces.

3- Improved selectivity towards intracellular and extracellular bacterial strains.

Utilizing liposomes as drug delivery agents showed tremendous results in eradicating intracellular strains via enhancing antibiotic retention in the infected tissues, providing controlled drug release with minimal toxic effects, and maximizing the concentration at the infected area. For targeting macrophage infections, anti-tubercular drugs loaded within stealth liposomes with small interfering ribonucleic acid (RNA) were fabricated [46]. The prepared system successfully inhibited the transforming growth factor-β1, eliminating the infection compared to the free drug.

For extracellularly multiplying bacteria, including *Pseudomonas aeruginosa*, weakness of inhaled antibiotic for curing *P. aeruginosa* infection accompanied with cystic fibrosis was reported due to poor drug permeation, inactivation by sputum, reduced efficacy against the protective biofilm, and shortened lung residence. Bilton et al. [47] investigated the potential of inhalation suspension of amikacin-loaded liposome (ALIS) and inhalation solution of tobramycin (TIS) in an open-label, randomized, phase III clinical trial. The findings confirmed the hypothesis that ALIS was similar to TIS for curing chronic *P. aeruginosa* infection accompanied with cystic fibrosis as shown from the comparable enhancements in forced expiratory volume in 1 s (FEV1%) and reductions in *P. aeruginosa* sputum density that were identical in the 2 arms.

• Limitations of antibiotics-loaded liposomes as drug delivery agents:

Despite the significant improvements in antibiotics delivery using liposomes, these lipid vesicles also suffer from many drawbacks limiting their efficient usage.

1- Physical and chemical instability problems, that can be minimized by addition of antioxidants and/or freeze-drying [28].
2- The possibility of antibiotic leakage from liposomes under physiological conditions, that can be controlled by adding cholesterol which lead to stabilization of liposomal membrane [48].
3- The low loading capacity of liposomes compromises the liposomal usage as antibiotic delivery agent. This challenge can be solved by maximizing electrostatic attractions between liposomes and oppositely charged antibiotic molecules [49,50].
4- Special sterilization techniques are needed due to the sensitivity of lipids to high temperatures [51].
5- Fabrication techniques are very complex, expensive, and difficult to be scaled up [52].

• Classification of liposomes:

Generally, liposomes have been categorized either based on their composition, vesicle size, bilayers number, and/or technique of preparation. In this context, the classification of liposomes according to their design and physicochemical characteristics into conventional, fusogenic, surface-modified, reactive liposomes encapsulating enzyme(s), antibiotic-metal co-encapsulating, liposomes-in-hydrogel, solid-supported liposomes, liposome-loaded scaffolds, and miscellaneous liposomes will be discussed [31].

1- Conventional Liposomes

Conventional liposomes are regarded as bare liposomes, lacking any surface modulations. They are made up of phospholipids with or without cholesterol addition. Based on the surface charge of the used lipids, they can be grouped into uncharged, negatively charged, or positively charged liposomes of which positively charged liposomes expressed dramatic improvements in biofilm targeting due to the electrostatic attraction with the anionic biofilm surface. Interestingly, Arikace™^®^ and Lipoquin™^®^ are two examples of conventional liposome preparations of amikacin and ciprofloxacin, respectively, which are used for cystic fibrotic patients with *P. aeruginosa* infections. Arikace™^®^ passed phases II and III of clinical trials and Lipoquin™^®^ passed a 14-day phase II trial, proving their tolerability, safety, improved biologic activity, and restoration of lung function [53,54].

2- Fusogenic Liposomes

Fusogenic liposomes are well famed as Fluidosomes™. They are distinguished by relatively soft lipid bilayers compared to the rigid conventional liposomes. The presence of special lipid (phosphatidyl ethanol amine) that renders the vesicles more fluid encourage the reduction of the membrane transition temperature and destabilize the lipid packing [55]. The enhanced anti biofilm activity of tobramycin Fluidosomes™ against many strains such as *B. Cepacia (Burkholderia cepacia), S. maltophilia (Stenotrophomonas maltophilia), P. aeruginosa, E. coli (Escherichia coli)*, and *S. aureus* at sub-MIC (minimum inhibitory concentration) levels were reported compared to the corresponding free antibiotic [56]. Furthermore, Beaulac et al. [57] elucidated the superior in vivo bactericidal activity of tobramycin loaded in the negatively charged Fluidosomes™ agianst *P. aeruginosa* infection.

To realize the mechanism of fluid liposomes interaction with bacteria, Wang et al. [58] realized that the bactericidal effect of tobramycin encapsulated fluid liposomes occur fast when bacteria is co-cultured with liposomes as a result of fusion process between liposomes and bacteria rather than the prolonged residence and release of antibiotic. This fusion process is dependent on degree of fluidity, temperature, pH, and presence of divalent cations as well as the properties of bacterial membranes.

3- Surface-Modified Liposomes

The application of surface-decorated liposomes, for example mannosylated liposomes, immune liposomes, and PEGylated liposomes, is among the proposed strategies for designing long-circulating liposomes to surmount biofilm-related infections. However, the strategy still suffers from many optimization challenges. Tatsuhiro Ishida et al. [59] revealed the loss of long-circulating features of PEGylated liposomes following their intravenous administration to a mice model as evidenced by accelerated blood clearance. Although, polyethylene glycol (PEG) coat prevented this loss to some extent. The observation was attributed to the degree of PEGylation and the amount of lipid. Therefore, further studies will be imperative in order to design effective liposomal preparation suitable for clinical application.

To further examine the effect of the PEG coat on the anti-biofilm activity of liposomes, PEGylated anionic and cationic liposomes were formulated and tested against *S. aureus* biofilms. Surprisingly, the results revealed the loss of anti-biofilm activity of liposomes after coating with PEG [60]. On the other hand, rifampin-loaded cationic liposomes either with PEG coat or without had the same anti-biofilm activity towards to *S. epidermidis* biofilm [61]. To explain this, the authors concluded the direct relation between incubation time and the anti-biofilm efficacy.

4- Reactive enzyme(s)-loaded liposomes.

The use of either one or more enzyme(s) loaded within liposomes represents one of the pioneer approaches in the field of anti-biofilm therapy. Moreover, encapsulation of enzymes within liposomes vesicles guarantees their adsorption and stay near to the biofilm surface. The antibacterial activity of reactive-enzyme(s)-loaded liposomes are governed by many factors, such as the enzyme entrapment efficiency, zeta potential, and phospholipid composition of liposomes [62].

For example, endolysins enzymes were successfully encapsulated within cationic liposomes. Contrary to free endolysins that have limited activity, only towards Gm +ve strains, and unable to cross the outer membrane of Gm–ve ones, endolysins entrapped within liposomes could successfully cross bacterial membrane and reach their target peptidoglycan substrate, showing significant reduction in logarithmic growth of live cells of *S. Typhimurium* and *E. coli* Gm–ve biofilms [63].

Another approach, which relies on the production of hydrogen peroxide or other oxidizing agents having antimicrobial properties upon contact of enzymes with certain substrate, was discovered. To test this, encapsulation of either a single glucose oxidase (GO) enzyme or coupled glucose oxidase-horse radish peroxidase (GO-HRP) enzymes within DPPC/PI liposomes was performed. The coupling of enzyme with glucose substrate leads to production of hydrogen peroxide, which yields oxy acids that have powerful antibacterial activities against oral Streptococcus gordonii biofilms. In addition, it was concluded that coupled enzymes containing liposomes were more effective than single enzyme formulation [64].

Similarly, Jones et al. [65] encapsulated chloroperoxidase and lactoperoxidase in combination with glucose oxidase enzymes within DPPC/PI liposomes. The reactive liposomes expressed significant antibacterial activity towards Steptococcus gordonii oral biofilm attributed to the reaction of hydrogen peroxide and oxyacids produced with glucose, chloride, or thiocyanate enzyme substrates.

5- Antibiotic-Metal Co-Encapsulating Liposomes

Certain metals, for instance gallium, bismuth, and bismuth-ethanedithiol, have shown promising antibacterial effects. Their activity period comes from affecting iron-metabolism, alginate expression, bacterial adherence, or interference of quorum sensing (QS) signaling and production of virulence factors [66,67,68]. Following this approach, bismuth-ethanedithiol included in a tobramycin-loaded liposome preparation was fabricated by Alhariri and Omri [69]. At sub-minimum inhibitory concentration (MIC), liposomes-loaded metal tobramycin formulation weakens QS signaling and reduces the production of virulence factors such as lipase, chitinase, and protease, compared to both free tobramycin or tobramycin-loaded liposome preparation. In vivo antimicrobial activity of metal-tobramycin incorporated liposome formulation in rats chronically infected with *P. aeruginosa* showed significant count reduction of *P. aeruginosa* in lungs.

6- Liposomes-hydrogel system

This policy involves the application of antibacterial-loaded liposomes after being incorporated within a suitable gel base to provide a unique and robust formulation. The hydrogel formulation maintains integrity of the liposomal structure, provides tunable release rate, better bioadhesion, and possibility of surface modification [70]. For the first time, tetracycline HCl and tretinoin-loaded liposomes prepared by the thin film technique were incorporated in carbopol-based gel. The findings revealed enhanced extended release behavior of both drugs with an average 55% release of two drugs up to 24 h. Antibacterial efficacy of the prepared liposome in gel towards *S. aureus* and *Streptococcus epidermidis* biofilms has been confirmed. Therefore, it is an effective alternative option for treating *Acne vulgaris* [71].

Hydrogels also offer capacity for prolonged release of antibiotics for infection control in wounds. Raj Kumar Thapa and colleagues developed collagen mimetic peptide tethered vancomycin-loaded liposomes hybridized to collagen-based hydrogels for the management of MRSA infections. The formulation achieved sustained antibiotic release and enhanced antibacterial efficacy with successful management of wound infection within nine days [72]. In addition, an injectable, antibacterial, and self-healing multifunctional drug delivery system composed of adhesive liposomes loaded with bone morphogenetic protein 2 (BMP-2) incorporated into PEG hydrogels was successfully developed. The system could be used for the treatment of bone cavity damage and reduces the risk of postoperative infections. The presence of silver ions in the adhesive liposome PEG gel system showed effective inhibition of *S. aureus* and *E. coli* pathogens [73].

7- Liposomes supporting solid (SSLs) and liposome-loaded scaffolds (LLSs)

Liposome-supporting solid (SSLs) delivery relies on the loading of antibiotic-liposomes on solid particles surfaces. In this regard, the applicability of gentamicin-liposomes loaded onto particles of the calcium sulfate was tested. In vivo antibacterial study revealed the significant improvement of gentamicin SSLs more than gentamicin-loaded calcium sulfate and non-adsorbed liposomal gentamicin due to better targeting ability to the infection site [74].

Targeting bacterial biofilm may be achieved by designing LLSs, whereas antibiotics containing liposomes can be further loaded onto artificial bone scaffolds. To validate this technique, gentamicin-sulfate liposomes have been impregnated onto beta-tri calcium phosphate granules. The in vitro release profile exhibited initial fast release of liposomal gentamicin from the scaffold matrix followed by more prolonged release of the free antibiotic from liposomes. The designed delivery system LLSs displayed significantly elevated anti-biofilm activity compared to free antibiotic [75].

8- Miscellaneous liposomes

Miscellaneous liposomes such as biomineral-binding liposomes (BBLs) were proposed for treating device-associated osteomyelitis and for delivering antimicrobials to the skeletal muscles efficiently [76]. The applicability of liposomes to develop antimicrobial surfaces for construction of efficient medical devices was also explored. Tobramycin-loaded liposomes were immobilized on gold-deposited stainless-steel surfaces and antibacterial efficacy was evaluated against *S. epidermidis* (*Staphylococcus epidermidis*) (American type culture collection; ATCC 35984 and ATCC 12228) strains. Antibiotic-liposome coated surfaces were found to possess good antibacterial activity especially for non-biofilm forming strains [77]. Examples on the recent published studies of different types of liposomal preparations used to eradicate bacterial biofilm infections are illustrated in Table 1.

##### Lipid-Based Nanoparticles

Solid lipid-nanoparticles (SLNs) and nanostructured lipid carriers (NLCs) represent the two main nanoparticles sub-types made up of lipids. SLNs are drug delivery systems in colloid form composed of high melting-stable lipids that were developed to beat the instability problems of liposomes [90]. Techniques for fabrication of SLNs include; solvent emulsification-diffusion, supercritical fluid, microemulsion-based, and film-ultrasound dispersion method [91].

SLNs are characterized by their nanosize range, thus bypassing uptake by reticuloendothelial system; provide high protective effect of incorporated drugs from degradation, offer great targeting, and controlled release opportunity. In addition to their biocompatibility and biodegradability, the possibility of easy scale up may be another advantage. However, their therapeutic application may be hindered by their reduced drug loading potential and possibility of drug ejection during storing [92].

In the war against resistant bacterial infections, researchers explored the idea that the bioavailability of many antibacterial drugs were enhanced upon their incorporation within SLNs, such as clarithromycin, rifampicin, tobramycin, and ciprofloxacin [93,94], in addition to many formulation patents reporting the oral use of SLNs loaded with anti-tubercular drugs [95,96].

Nanostructured lipid carriers (NLCs) are considered as the advanced type of SLNs. NLCs composed of a rigid matrix blended with a liquid oil to form an unstructured matrix. Unlike SLNs, they form an imperfect core and an amorphous matrix for better drug loading ability and minimized drug escape from the matrix during storing [97]. In addition to their high loading capacity for both hydrophilic and lipophilic therapeutics is their capability to pass through multiple biological barriers and efficiently deliver the enclosed therapeutic moieties [98]. Furthermore, NLCs can be fabricated to be stimulated by various parameters such as pH and light for controlling the drug release [99,100]. However, the literature on the usage of NLCs for delivering antibacterial drugs is limited (Table 2). Therefore, there is an imperative requirement to further examine this system for improving antibacterial delivery.

##### Polymeric Micelles

A typical self-assembled micellization procedure involves the attachment of a hydrophilic block to a lipophilic block resulting in formation of micelles of amphiphilic di-block (or multiple block) copolymers with some characteristic structural properties that differ from either parent. Common polymeric backbones used for constructing micellar systems include chitosan, polyethylene oxide, polypropylene oxide, polybutylene oxide, polystyrene oxide, polyethyleneimine, and polycaprolactone. The size of polymeric micelles is from almost 10 to 100 nm [109].

The polymeric micelles size may be easily controlled by varying the molecular weight and the aggregation number of the amphiphiles, the ratio between hydrophilic and hydrophobic parts, the volume of solvent trapped within the core, and the used technique for preparing [110]. Using polymeric micelles as antibiotic delivery systems can dramatically enhance the pharmacokinetics and biodistribution of entrapped antibiotics. Micellar nanocarriers have the capacity to load poorly water-soluble drugs into their cores [111], better protection of incorporated antibiotics from destructive enzymes, prevention of drug interaction with blood proteins, thus keeping its appropriate plasma concentration.

More recently, for better targeting efficiency, stimuli dual-responsive polymeric micelles have been explored. For instance, poly (N-iso propyl acrylamide) is a commonly used temperature-responsive polymer. However, most studies on dual responsive polymeric micelles were limited to anticancer drugs with very scarce reports on antimicrobials [112,113]. The different pharmaceutical applications of antibacterial polymeric micelles are summarized in Table 3.

##### Polymeric Nanoparticles

Polymer-based nanosystems used against antibacterial resistance can be categorized into polymers that have themselves antibacterial properties and polymeric nanoparticles acting as antibiotic delivery systems [7]. Polymer nanoparticles are among the class of organic macromolecule-based antibacterial drug carriers that have many advantages such as ease of fabrication, physical and chemical stability in physiological environment and under storage conditions, easily controllable physicochemical properties, and prolonged drug release with better targeting efficiency [123].

Chitosan was proved to be the most efficient and versatile polymeric material from a natural source utilized for preparing antibacterial loaded drug carriers. The antibacterial activities of chitosan are dependent on numerous factors such as chitosan deacetylation degree and molecular weight, as well as the chemical structure and functionalization of chitosan molecules [124]. There are variable mechanisms that illustrate the antibacterial activity of chitosan, most commonly depending on the electrostatic attraction between chitosan and anionic surface of bacteria that lead to changing the permeability of cell membrane. Leaking out of the bacterial components results in cell death [125]. Examples of recent innovative studies done on antibiotic loaded chitosan nanoparticles are shown in Table 4.

Other examples of natural polymers include dextran sulfate and chondroitin sulfate polysaccharides. Nanoparticles of either chondroitin sulfate or dextran sulfate were formulated with high encapsulation efficiency around 65% and size ranged from 100 to 200 nm. The results indicated that macrophages intracellular uptake of the antibiotic by using antibiotic-loaded dextran sulfate nanoparticles was 4-fold that of the antibiotic-loaded chondroitin sulfate nanoparticles. Further, enhanced anti-microbial activity against intracellular salmonella infections was confirmed [126].

The main merits of natural polymers are being highly biodegradable and biocompatible. On the other hand, aforementioned merits may be originated in synthetic polymers too, for example PLGA (poly (lactic-co-glycolic acid) or PCL) (poly (*ε* -caprolactone)). PLGA is a commonly used synthetic polymer. Recently, it has been utilized as basic excipient for antibacterial polymeric nanoparticles production [127]. Clarithromycin antibiotic was successfully encapsulated within PLGA particles. The enhanced antibacterial efficacy against *H. pylori* strains was evidenced from lower MIC values compared to free antibiotic. Although the mechanism remains unclear, it was postulated that PLGA-loaded nanoparticles could carry out either fusion or adsorption [128]. PCL can be utilized for producing good antimicrobial drug delivery nanosystems because of its biocompatibility and biodegradability [129]. For instance, a significant improvement of anti-tubercular rifampicin uptake into macrophages was observed after its encapsulation within PCL nanoparticles compared to free drug, thus improving its antibacterial efficiency towards *M. tuberculosis* infection [130]. Recent literature on the application of polymeric nanoparticles against bacterial infections is described in Table 4.

#### 2.2.2. Inorganic Nanosystems

##### Silver NPs (Ag-NPs)

Nano-silver is known to possess a strong bactericidal activity against a variety of bacteria. The remarkable antibacterial action of Ag-NPs is a result of their well-expanded surface that provides the highest contact with the bacterial membrane leading to cellular leakage and cell growth inhibition. In addition to their binding affinity to macromolecules, their contact with the bacterial membrane lead to disintegration of the bacterial cells and death.

Several literatures reported the characteristic antibacterial activity of silver nanoparticles [146]. The activity of silver nanoparticles synthetized from the extract of *Corchorus Capsularis* leaf was evaluated against coagulase negative staphylococci, *P. aeruginosa* isolates of post-surgical wound infections, and *S. aureus (Staphylococcus aureus)* [147]. The results revealed that there was an inverse relationship between the concentration of silver nanoparticles and the number of bacterial cells. In addition, increasing time of bacterial exposure to Ag NPs resulted in lower survival of bacterial cells.

The extract of Trichodesmium erythraeum was utilized for preparing silver nanoparticles [148]. The diameter of prepared nanoparticles was 26.5 nm. The antibacterial findings displayed remarkable inhibition against *S. aureus* and *Proteus mirabilis* strains with minimum zone of inhibition 11 mm and 10 mm, respectively, and against antibiotic-resistant strains, for example *S. pneumonia* (PenicillinR) and *S. aureus* (TetracyclineR). Another synthesis technique for Ag-NPs by gamma irradiation was also reported by Swaroop et al. [149]. *E. coli* culture supernatant was also used for biosynthesis of silver nanoparticles. The fabricated particles exhibited a characteristic shape with mean size 33.6 nm. The formulated Ag NPs showed zone of inhibition of 13, 11, 10, and 10 mm, against *K. pneumonia, P. aeruginosa, E. coli*, and *S. aureus*, respectively.

Antibacterial activity of Ag-NPs was remarked against *E. coli*, *K. pneumonae*, and *P. aeruginosa*. These findings was also accompanied with decrease concentration of polysaccharides, lipids, proteins, and nucleic acids in biofilm compared with controls [150]. The antimicrobial action resulting from conjugating cationic peptides on the surface of either gold or silver nanoparticles was evaluated [151]. The findings indicated that silver nanoparticles surface decorated with cationic peptide revealed higher antimicrobial efficiency relative to peptide decorated gold nanoparticles as well as undecorated metallic nanoparticles and native peptides. Unfortunately, the use of silver and gold nanoparticles is limited on a large scale because of their high outlay of the production.

##### Silica Nanoparticles

Mesoporous silica nanoparticles have proven to be the answer in the management of diverse infections, as these nanoparticles have merits at all treatment phases, such as drug release, targeting biofilm, and adjuvant capacity [152]. Levofloxacin enclosed within nanoparticles of mesoporous silica capped with the lectin concanavalin A were formulated for the management of bone infections. The results interestingly showed that samples treated with bare silica nanoparticles presented a little activity towards biofilm. However, after treatment with levofloxacin-loaded silica nanoparticles, the biofilm reduction was more visible, due to the action of antibiotic.

The adsorption of certain biocidal on the surface of silica nanoparticles was evaluated by Jang et al. [153] who reported the fabrication of silica–poly(3-allyl-5,5-dimethylhydantoin-co-methyl methacrylate) (poly(ADMH-coMMA)) core–shell nanoparticles as a biocidal polymeric agent using a seeded polymerization. The produced mixtures (ADMH–MMA) were used to decorate the surface of silica nanoparticles, as a result of their hydrophobic properties, to produce cyclic N-halamines, which are utilized as antimicrobial agents. These N-halamine-decorated silica-nanoparticles showed excellent antibacterial action against both Gram-negative bacteria and Gram-positive bacteria, and their antibacterial actions have been efficiently enhanced compared with their bulk counterparts [154].

##### Magnetic Nanoparticles, MNPs

Magnetic nanoparticles have received widespread use in the field of biomedical and nanomedicine [155]. The antibacterial activity of MNPs are because of their damaging effect to the bacteria through interfering with the thiol group at the respiratory base of the bacteria [156].

The antibacterial efficacy of biosynthesized MNPs was tested against various drug resistant bacteria, such as *E. coli, Shigella, P. aeruginosa, S. aureus, Salmonella typhi*, and *Pasteurella multocida*. Agar-diffusion method confirmed the efficiency of magnetic nanoparticles to suppress the growth of *S. aureus* and *E. coli* in a concentration-dependent manner. Moreover, it displayed strong efficacy against all bacteria when compared with the standard drugs [157].

Magnetic nanocomposites were synthetized via sol-gel method. The sizes of the formed nanocomposites were in the range of ~71–91 nm. Antimicrobial activity of nanocomposites was studied against different bacterial and fungal pathogens. Minimum inhibitory concentration and the minimum bactericidal concentration values were observed within the range of 256–2048 µg/mL against *E. coli* and *S. aureus*. As expected, they could inhibit the growth of Gram-positive strain more effectively than Gram-negative strain. The existence of additional outer cell membrane renders Gram-negative bacteria more resistant to antimicrobial agents. Metal-ion release, reactive oxygen species generation, outer membrane and cell wall destruction, and particle internalization into microorganisms are the mechanisms of inhibitory action of metal oxide NPs on bacteria and fungi [158].

##### Zinc Oxide (ZnO) Nanoparticles

The U.S. Food and Drug administration has approved ZnO as a safe material. Many recent researches focus on ZnO, utilizing it as an antibacterial agent [159]. Antibacterial action of zinc oxide nanoparticles against various human pathogens was evaluated. The results revealed that zinc oxide nanoparticles displayed enhanced activity against *S. aureus* and low efficacy against Mycobacterium bovis-BCG (Bacillus Calmette–Guérin). The mechanism of ZnO-NPs as antibacterials relies on their ability to damage integrity of the cell membrane, diminish hydrophobicity of the cell surface, and down-regulate certain genes in bacteria. Moreover, ZnO-NPs treatment enhanced the intracellular bacterial damage by producing reactive oxygen species. Furthermore, ZnO-NPs prevents biofilm formation and hemolysis by hemolysin toxin that is produced by *S. aureus* [160].

It is worth to note that the antibacterial action of these metal oxide nanoparticles significantly relies upon their size. The size is important crucial factor because of entrance ease of small-size particles through pores of the bacterial cell surface. These pores in the bacterial cell surface are in nanometer size range. Additionally, ZnO nanoparticles exhibited an anticancer activity as compared with normal cells. Two mechanisms were predicted based on producing reactive oxygen species (ROS), toxicity of ZnO, and inducing apoptosis [161].

##### Cobalt NPs

Cobalt oxide NPs are receiving wide spread attention lately due to their structural, antibacterial, and biomedical activities [162]. Satpathy et al. [163] evaluated the antibacterial characteristics of cobalt NPs towards isolated *E. coli* strains. The results confirmed the dependency of antibacterial effect of cobalt nanoparticles on both particle size and cobalt concentration as reflected by the enhanced bactericidal effect of cobalt nanoparticles (35 µg/mL, 200 nm).

These results were contradictory to Khan et al. [164] who examined the anticancer and antibacterial action of cobalt oxide nanoparticles (Co_3_O_4_-NPs) on cancerous cells of colon and on bacteria, respectively. The results revealed that Co_3_O_4_-NPs displayed anti-cancerous characteristics. However, no antibacterial action was reported. Khalil et al. [165] also evaluated the antibacterial characteristics of Co_3_O_4_-NPs either illuminated or without UV illumination. It was found that on UV illumination, antibacterial characteristics of Co_3_O_4_-NPs were improved, confirming the antibacterial potential of cobalt oxide NPs against different bacterial strains.

Further, Dogra et al. [166] fabricated nanosuspensions of metallo surfactants-derived cobalt oxide and hydroxide via the microemulsion method. The results revealed that nanosuspension made up of (Bis hexa decyl trimethyl ammonium cobalt tetrachloride) surfactant has maximum antimicrobial activity against multiple-medicine-resistant *S. aureus*. Cell shrinkage, formation of holes, change of morphology, and cell wall rupturing was observed.

##### Selenium Nanoparticles (Se-NPs)

Selenium is one of the trace essential elements that recently attracted attention due to its antitumor actions, importance in the immune system, and effect on certain hormones such as thyroid hormones [167,168]. To evaluate the antibacterial activity of selenium, Yazhiniprabha and Vaseeharan evaluated the bacteriostatic and larvicidal efficacy of biosynthesized Se-NPs. The results displayed a significant larvicidal property against the fourth instar larvae of a dengue fever-causing vector *Aedes aegypti* and anti-bacterial activity against Gram-positive (*Enterococcus faecalis* and *Streptococcus mutans*) and Gram-negative (*Shigella sonnei* and *P. aeruginosa*) bacteria at 40 and 50 μg/mL. In vitro and in vivo toxicity assessment of nanoparticles showed low cytotoxicity against (RAW 264.7) macrophages and *Artemia nauplii*. Thus, selenium nanoparticles can be proposed as a biocompatible nano-biomedicine against bacterial infections [169].

##### Cadmium Nanoparticles (CdO-Nps)

Owing to the ionic nature, stability, biocompatibility, and monodispersity of cadmium nanoparticles (CdO-Nps), all these properties make them good candidates to be used for delivering antibacterial drugs [170]. Zahera et al. [171] synthesized a highly biocompatible, monodispersed, and stable glucose capped CdO-Nps utilizing a sol-gel technique and compared it with naked CdO-Nps. The lowest inhibitory concentrations of glucose-capped CdO-Nps and naked CdO-Nps were 6.42 and 16.29 μg/mL, respectively, against *E. coli* strain, and 7.5 μg/mL and 11.6 μg/mL, respectively, against *S. aureus* bacteria. Glucose capping imparted stability and monodispersity to CdO-Nps, in addition to improved biocompatibility and penetrability into the living cells.

Although nanosystems possess immense potential as antibacterial delivery agents, different strains of bacteria display various levels of susceptibilities to different nanosystems. Various formulation factors for example the fabrication technique, particle size, and nanosystems composition, should be optimized in order to get the desired results.

##### Advantages and Drawbacks of Inorganic Nanoparticles

Inorganic-based nanosystems composed of inorganic metals, among which are silver (Ag), Silica, magnetic metals, selenium (Se), cobalt, and zinc oxide (ZnO), have been demonstrated with pronounced antibacterial activities [160]. However, very limited information is available on the in-vivo antibacterial efficacy of metal oxide NPs, their ability to kill pathogenic strains, and mechanisms of action. In general, metal nanoparticles have some advantages, such as large surface area and multimodal applications. However, toxicity, instability, and storage are major drawbacks of inorganic nanosystems [27].

## 3. Novel Approaches for Combatting Antibacterial Resistance

**Figure 2:**
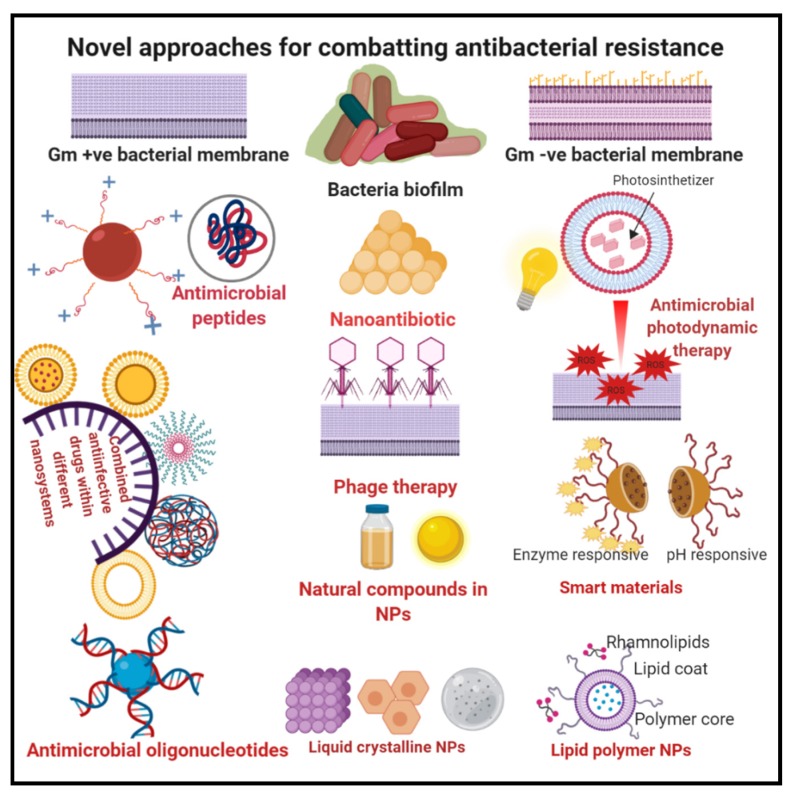
Diagrammatic illustration of the novel approaches utilized for combating antibacterial resistance.

In light of the abovementioned literature studies, we can conclude that there are many factors contributing to the biofilm resistance to antibiotics; consequently, the management based on only one parameter may not be enough for eradicating biofilm. An illustration of the recent novel approaches used to overcome antibacterial resistance, presented in Figure 2, will be discussed in the following section.

### 3.1. Lipid Polymer Nanoparticles (LPNs)

In view of the unique and complex features of biofilm, it was hypothesized that a technique that directly damages the extracellular matrix of biofilm and subsequently causes death of the bacteria could perform an amelioration in biofilm elimination. Disruption of the biofilm matrix leads to release of bacteria that regain their susceptibility to the action of antibiotics. In addition to this is the possibility to inhibit recurrence of infection because planktonic bacteria could not re-adhere to guest cells [172].

Following this principle, the conjugation of rhamnolipid, which is a biosurfactant fundamentally secreted by *P. aeruginosa* [173], to polymeric nanoparticles was proposed by Li et al. [174] to overcome Helicobacter pylori biofilm resistance. These novel particulate systems are composed of chitosan polymer as the core, encapsulating clarithromycin antibiotic, and the shell is made up of mixed lipids containing (1,2-distearoyl-sn-glycero-3-phosphoethanolamine-N-[amino(polyethyleneglycol)-2000) DSPE-PEG_2000_-decoratedrhamnolipids. The fabricated particles have acceptable particle size ranging 148.5–165.2 nm and high clarithromycin encapsulation efficiency (>86%). The eradicating ability was observed to be remarkably enhanced as the lipoidal composition of rhamnolipid increased, which is represented by the considerable reduction of biofilm biomass and viability. These findings can be explained based on the disruptive power of rhamnolipid on biofilm matrix, the characteristic antibacterial properties of clarithromycin and chitosan NPs, the preventive actions of chitosan NPs and rhamnolipids on bacteria adhesion, and biofilm formation. Similarly, rhamnolipid-coated metallic nanoparticles (silver and iron oxide) were fabricated by Khalid et al. [175] and demonstrated excellent anti-biofilm efficacy against *S. aureus* and *P. aeruginosa* biofilms.

### 3.2. Nonlamellar Lyotropic Liquid Crystalline Nanoparticles

Various kinds of self-assembled nonlamellar liquid crystalline nanoparticles have been recently evaluated for their potential as drug delivery systems for antimicrobial molecules [176]. The system consists of amphiphiles that orient themselves into different mesophase structures, for example hexagonal, lamellar, cubic, and the less common sponge structure [177,178]. Advantages of these nanosystems include their amphiphilic nature and their larger interfacial surface areas, which enable them to encapsulate both hydrophilic and hydrophobic drugs, in addition to the ease of production with the ability to scale-up.

To test the antibacterial efficacy of this system, neutral and positively charged monoolein lipid nanoparticles encapsulating rifampicin were prepared by Tran et al. [179]. The results explained the highest potential of cationic nanoparticles reflected by significant reduction of the minimum concentration required to inhibit growth of *S. aureus* compared to the use of rifampicin alone, suggesting that the bacterial membrane with negative charges are firmly electrostatic and interacted with the lipids with positive charges.

### 3.3. Anti-Microbial Oligonucleotides

Use of oligonucleotide in antibacterial therapy, for example transcription factor decoys (TFD), is considered as a hopeful policy to overcome antimicrobial resistance. Transcription Factor Decoys (TFD) are short fragments of DNA that act on specific genome by capturing certain regulatory proteins to stop essential genes in the bacterial cells and overcome infection [180]. However, finding a suitable carrier that offer DNA encapsulation and protection against nucleases with efficient targeting to infection site is a challenge. Gonzalez-Paredes et al. [181] investigated the possibility of use of anionic solid lipid nanoparticles that were coated with either the cationic bola amphiphile 12-bis-tetrahydroacridinium or with protamine as a suitable carrier for TFD. Both compounds shifted zeta potential to positive values and demonstrated protective effect of TFD from deoxyribonuclease enzyme and, hence, a preferred accumulation of TFD in bacteria.

Furthermore, in an attempt to solve the challenges of preserving the colloidal stability and transfection efficiency of TFD, Mamusa et al. [182] designed phosphatidylcholine/phosphatidylethanolamine- cationic bolaamphiphile12-bis-THA scaffold to form TFD-loaded cationic liposomes. Although high entrapment efficiency of TFD (about 90%) was achieved with a maximum protection from serum nucleases, further studies towards a pre-clinical preparation against antimicrobial-resistant infections still need to be evaluated. Many authors also reported the possibility of conjugation of oligonucleotide antimicrobials with cationic materials such as peptides for penetrating cell (CPP). However, utilization of CPP is restricted to neutrally charged oligonucleotides, otherwise precipitation will occur [183]. Although these conjugates showed marked performance in an in vivo and in vitro bacterial patterns, the concentration used was relatively high, limiting their application due to cytotoxicity hazards [184].

Recently, strategy of oligonucleotide therapeutics was applied for acting on new therapeutic targets, for instance transcription, however due to undesirable physicochemical characteristics such as size, charge, and hydrophilicity that limit effective permeation across bacterial membranes to reach their target site, new strategies should be developed to solve the problem of efficient delivery of oligonucleotide therapeutics [185].

### 3.4. Combination of Nanotechnology and Natural Compounds

Inclusion of natural components in nanoparticles fabrication have recently been encouraged to change the carrier matrix and boost the antibiotics efficacy [186].

For instance, Rodenak-Kladniew et al. [187] examined the effect of incorporation of chitosan and eugenol, a natural phenolic compound, into a lipid matrix containing ofloxacin antibiotic by hot homogenization/ultrasonication technique. The results indicated that the developed formulation displayed an improved bactericidal action against *P. aeruginosa* and *S. aureus* significantly. Eugenol is known to induce nanostructuration of the matrix, enhance the particles stability, and conflict with bacterial growth, in addition to its high skin penetrability.

### 3.5. Smart Materials

Among the newly discovered nano antibiotic delivery platforms is the utilization of smart moieties, for example pH [188] and enzyme [189] responsive materials, for targeting bacterial infection tissues. pH-triggered antibiotic release achieves increasing importance nowadays. Designing polymers with pH-responsive materials, for example poly-l-histidine [190], comes with the possibility of pH triggered surface charge switching in acidic medium of the infected bacteria. This specific feature is utilized to maximize the attraction force to the bacterial cell wall having negative charge and hence release the antibacterial drug more precisely in the targeted site.

### 3.6. Cationic Peptides

One of the hopeful techniques to combat bacterial resistance is the inclusion of cationic peptides as an option to conventional antibiotics [191]. The unique features of cationic peptides include their amphiphilic nature and their cationic charge, which facilitate targeting of negatively charged bacterial membrane leading to escape of intracellular contents and death [192]. Further, due to the impossibility in restoration of the damaged cell structure, this efficient strategy minimizes the emergence of bacterial resistance.

### 3.7. Antimicrobial Photodynamic Therapy (aPDT)

Utilization of phototherapy for killing microbes is considered as a novel non-invasive treatment of choice to manage infectious diseases. The mechanism of photodynamic therapy depends on the local or systemic use of oxygen, combined with a photosensitizer (PS) and a visible light, producing reactive oxygen species (ROS) upon exposure. Oxygen species cause oxidative stress to ingredients of the bacterial cells such as membranes leading to complete biofilm elimination [193]. The unique advantage of this technique over other conventional ones is the dual selectivity behavior. The photosensitizer only accumulates in the rapidly growing bacterial cells and application of light leads to the photo-destructive effect limited to the area where the light is delivered, criteria that prevent the recurrence of infection [194]. Sanjana Ghosh et al. [195] investigated the loading of ciprofloxacin into photoactivatable liposomes that were made of porphyrin-phospholipid. The authors reported that about 90% of the antibiotic released in less than 30 s. Moreover, with or without laser treatment, ciprofloxacin photoactivatable liposomes inhibited the growth of *Bacillus subtilis* in liquid media, probably due to the enhanced uptake of liposomes by bacteria. These findings show the feasibility of photoactivatable liposomes to enhance localized antibiotic therapy.

Synergistic antibacterial effect was obtained by coating pullulan (PL)/pheophorbide-A (phA) conjugates (PL/phA) onto erythromycin-loaded liposomes. *P. acnes* skin infections characterized by secreting lipase enzyme that disrupt erythromycin-loaded liposomes led to the release of encapsulated drug and PhA conjugate. Laser irradiation onto the liberated PhA leads to the maximum *P. acnes* growth suppression and the curing of *P. acnes*-infected inflammation [196].

### 3.8. Nano Systems with Combination Drug Therapy

The gradual back off in mono-drug therapy, due to the resistant strain’s emergence, made the combination drug therapy a first-line option for better outcomes. The principle of combined drug therapy relies on the simultaneous use of multiple therapeutics called a “drug cocktail” to treat bacterial infections with the goal of achieving synergistic drug effects, combating resistance, minimizing side effects, and expanding antimicrobial spectrum [197,198]. Moreover, the co-encapsulation of the combined drugs within nano-systems will offer advances in managing resistant bacterial infections, however the practical application is still in preliminary stages [199]. Fabrication of liposomal formulation co-encapsulated with ciprofloxacin and colistin was done in order to treat *P. aeruginosa*-induced respiratory tract infections. The in vitro antibacterial findings displayed that the prepared co-loaded liposomes had better anti-*P. aeruginosa* efficacy than the monotherapies, in addition to improved retention time and prolonged release of the encapsulated drugs at the infection tissues on the lung. However, in vivo evaluations are required to assure the applicability of the system to treat multi resistant respiratory infections [200].

In line with this approach, co-encapsulation of aminoglycoside antibiotic (tobramycin) with macrolide antibiotic (clarithromycin) within proliposomes with the aim of synergistic management of respiratory infections caused by *P. aeruginosa* was investigated. High entrapment efficiencies of both hydrophobic clarithromycin (89%) and hydrophilic tobramycin (47%) were obtained. The results showed synergistic antimicrobial activity against in vitro *P. aeruginosa* biofilms compared to either drugs alone [201]. Moreover, the co-encapsulation of LL37 and serpin A1 in solid lipid nanoparticles showed synergistic effect towards *E. coli* and *S. aureus*. for treatment of wound infections, although the mechanism of synergism was unknown [202].

### 3.9. Nano-Antibiotic

Nano-antibiotic is a promising technique where the transformation of the therapeutic agents themselves into nano-sized assemblies can be done, thus considered as carrier-free drug delivery approach. This approach is of great interest due to the fact that it can modify the physical properties of antibiotics, increase their dissolution rate, improve drug bioavailability, reduce side effects, better contact with microorganism, improve interaction and penetration within bacterial membrane, thus perform better against antibiotic-resistant strains [203].

Morakul et al. [204] investigated the potential of clarithromycin nanocrystals towards Helicobacter pylori infections. The formed nanocrystals enhanced the bioavailability and availability of drug at the desired site of action as compared to the lyophilized coarse suspension and the clarithromycin powder. More recently, using hyperbranched polyesters themselves as a new form of nano-grade antibiotics alleviating the complications of antibiotic encapsulation and release was explored [205].

### 3.10. Phage Therapy

Phage therapy is considered as a safe and effective technique against resilient pathogens. However, up to date, none of the phage therapies have successfully extended its application to the consumers. The major drawbacks that hinder the application of this strategy include high specificity and poor pharmacokinetic properties [206].

In an attempt to overcome the narrow host range and rapid clearance drawbacks, Chadha et al. [207] has demonstrated the potential of phage cocktail loaded-liposomes rather than the monophage therapy and tested it against *K. pneumoniae* inducing burn wound infections. The findings of the study confirmed the better reduction of bacterial load in main organs and blood of the infected mice treated with liposomal entrapped phage cocktail as compared to non-liposomal free phage cocktail. Additionally, liposomal phage formulation was able to save all the examined animals from death even when there was a slowness of starting the therapy for 24 h. In spite of an increasing number of clinical trials confirming the activity and safety of phage therapy, there are still missing regulatory information that require to be handled before phage therapy can be applied broadly.

## 4. Clinical Trials

### 4.1. Current and Future Market of Nanosystem Antibiotics

After these extensive research efforts in developing innovative antibacterial delivery systems to resist the antibiotic resistance crisis, the good news is, there are number of nanosystem-based antibiotics, antitoxin agents, and antimicrobial peptides that have been recently translated to the clinic. However, many are still in different stages of clinical trials (Table 5 and Table 6).

#### 4.1.1. Antibiotic Agents

The first clinical application of inhaled ciprofloxacin-loaded liposome using Lipoquin was a phase 1 trial in certain healthy volunteers [54], as shown in Table 5. Then to evaluate activity, initial safety, and pharmacokinetics of once-daily inhaled ciprofloxacin-loaded liposome (Lipoquin), a Phase 2a multi-center 14-day trial was conducted in 21 adult CF patients. Concurrently, an international, double-blind, randomized, phase 3 trials (ORBIT-3 and ORBIT-4) were run in similar regions to investigate the safety and efficacy of inhaled liposomal ciprofloxacin [208,209]. Moreover, amikacin-loaded liposomes were evaluated in many clinical studies. In a double-blind, phase 2, randomized study, efficacy, safety, and tolerability of once daily dosing of amikacin 590 mg versus placebo for 84 days were explored in subjects with treatment refractory Nontuberculous Mycobacteria lung infection on a stable multidrug regimen [210]. Another study (phase 2) investigated the long-term efficacy, safety and tolerability of once daily 560 mg dose of inhaled amikacin-loaded liposome, administered for six cycles over 18 months, in cystic fibrosis patients with chronic infections caused by *Pseudomonas Aeruginosa* [211].

A new study of phase 2 trial will assess the efficacy, safety, and tolerability of inhalation liposomal amikacin, once daily dosing for 12 months of 590 mg plus standard-of-care mycobacterial multi-drug regimen, for treatment of mycobacterium abscesses lung disease [212]. In a phase 3 clinical trial, cystic fibrosis patients with chronic infection due to *Pseudomonas aeruginosa* were included in the study and the long-term tolerability and safety of inhaled amikacin-loaded liposome (590 mg/day) was studied [213]. Various nanosystem-based antibiotics and anti-toxins in clinical trials different stages were listed in Table 5.

#### 4.1.2. Anti-Toxin Agents

New nano-preparation targeting bacterial components responsible for virulence effect of bacteria, such as toxins, may be a promising challenge in the area of antibacterial medications. Bezlotoxumab was the first antitoxin, approved in 2016, as a human monoclonal antibody targeting toxin B of Clostridium difficile [214]. A certain number of anti-toxin agents such as monoclonal antibodies targeting S. aureus’ α-toxin are in the clinical development, in addition to monoclonal antibodies that target type III toxins secretion moiety of *P. aeruginosa* [215]. A broad-spectrum antitoxin liposomal agent (novel empty liposomes is also in clinical development; CAL02) has resulted in synergistic actions with drugs or antibiotic and also proved the ability to redeem mice from serious infections, for example staphylococci by adsorbing toxins [216].

#### 4.1.3. Antimicrobial Peptides

Antimicrobial peptides exhibit broad-spectrum antibacterial activity with relatively low risk of resistance development due to the rapid onset of killing [217]. The principle target of antimicrobial peptides is the cell membrane of bacteria. Other intracellular targets have been reported including synthesis of cell wall (nisin), synthesis of nucleic acid, synthesis of RNA (e.g., buforin II), enzymatic activity (pyrrhocoricin), protein synthesis (indolicidin), or ATP (Adenosine triphosphate) efflux (histatins) [217]. Many antimicrobial peptides currently undergoing preclinical and clinical trials are listed in Table 6.

## 5. Challenges in the Clinical Translation of Nanomedicine

**Figure 3:**
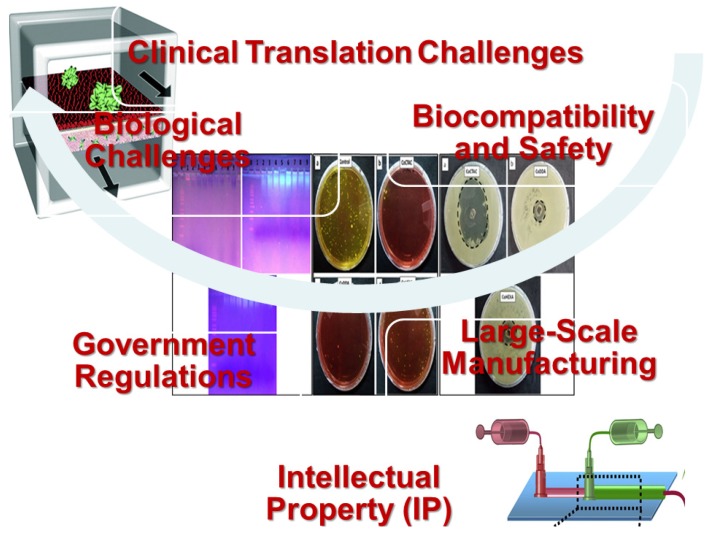
Graphical representation of the challenges in the clinical translation of nanomedicine.

Clinical translation of nanomedicine has difficulties, such as being time consuming and expensive. The main challenges related to the clinical translation are biological issues, safety, biocompatibility, intellectual property (IP), laws and regulations, and overall cost-effectiveness compared to traditional therapies [233], (Figure 3). These obstacles limit the usage of nanoparticles in the present markets regardless of their effectiveness [234]. Many issues should be considered during the clinical translation of nanomedicine. The first one is the nanopharmaceutical design that can be enumerated as follows; physical and chemical stability, biodegradability, sophisticated formulation design, and administration route. Efforts should be utilized for resolving obstacles of large-scale production, such as reproducibility and high cost, and also obstacles of quality control assays for characterization such as polydispersity, scalability complexities, incomplete purification from contaminants, consistency and storage stability of the final product, morphology, and charge [235,236]. Preparation techniques are required to consistently produce large scalable quantities of nanoparticulates with high degree of quality and batch-to-batch reproducibility.

The second issue is preclinical evaluation, such as the need for early detection of the toxicity, in vivo evaluation in appropriate animals, and understanding both pharmacokinetics and pharmacodynamics. Therefore, more advanced toxicological evaluations for nanomedicine should be developed. Moreover, stability of nanoparticulates following administration and interaction of nanoparticulates with tissues should be well understood. The third issue concerns clinical examination for commercialization. Pathways from invention to the markets are complicated, so they should be minimized to save the time and the cost. Additionally, safety/toxicity in humans and therapeutic efficacy in patients should be evaluated using more simple techniques. Specialized toxicology evaluations in animals should be carried out to examine both short-term and long-term toxicity, as biological half-lives are significantly raised with nanoencapsulation.

Clear regulatory guidelines specific for nanoparticulates should be developed for sensitive, validated, and standardizable examinations incorporating protocols to appropriately evaluate the nanotoxicology of nanoparticulates during the early steps of clinical development [237]. Because nanoparticulates represent various types of nanostructures, there are challenges in developing regulatory protocols, techniques, and tools for ensuring standardized good manufacturing practice (GMP) production and characterization, safety, and economic design of clinical trial. Nanoparticulates patents and intellectual properties (IP) are complex and simplification of them is needed to simplify the pathway from invention to commercialization to reduce the time and expense required for negotiating collaboration and licensing agreements [238].

## 6. Recommendations and Perspectives

As noted, it becomes clear that translation of antibacterial-loaded nanosystems to the clinic is still beyond reach for most approaches, so more research efforts became imperative to solve the massive challenge of antibiotic resistance. The author recommendation is that, presently, the community now has another task, which is to “do more efforts for clinical translation of nanoparticulates and takes their advantages in a more comprehensive way’’. Successful clinical translation firmly demands an interdisciplinary strategy to develop creative protocols, examinations, and infrastructure for large-scale manufacturing of nanoparticulates. Therefore, we invite all academic and pharmaceutical industry experts with specialty in medicine, biology, pharmaceutics, toxicology, and engineering to undertake more efforts to overcome these challenges.

Prospective techniques to fast-track favorable nanoparticulates to clinical applications encompass the coordination of faculties, pharmaceutical factories, and laboratories that have excellent expertise in evaluating nanoparticulates platforms, performing preclinical studies, and designing and carrying out clinical trials of nanoparticulate platforms [239].

## 7. Concluding Remarks

As defeating bacterial resistance growth is very costly and time consuming, due to high cost of the process of production of new safe and effective antibiotic drugs, new policies for controlling infections became requisite. The recent strategies displayed in this manuscript were based on using nanosystems to surmount antibiotic resistance. In summary, nanosystems are classified based on their matrix composition into organic nanosystems (liposomes, lipid-based nanoparticles, polymeric micelles, and polymeric nanoparticles) and inorganic nanosystems (silver, silica, magnetic, ZnO, cobalt, selenium, and cadmium). The antibacterial activities of either free nanosystems or their synergistic effects when loaded with conventional antibiotic molecules were reported. The physicochemical properties of these nanosystems, for example zeta potential, particle diameter, and solubility, are the important parameters in their employment as antibacterial drug delivery systems. As explained in this context, various mechanisms by which nanosystems were able to overcome antimicrobial resistance were listed, as follows; offer high entrapment efficiency of either hydrophilic or lipophilic drugs, protection of entrapped antibacterial drugs from bacterial enzymatic inactivation, the potential of nanosystems to target the site of infection, increased uptake or decreased efflux, and physical damage of the cell membrane, thus inhibiting re infection.

As reported in the literature, the results of using antibacterial loaded nanosystems against biofilm infections revealed a significant reduction in MIC values compared to their corresponding free unloaded drugs, which have great benefits of delaying or inhibiting the resistance development. More importantly, the review article reported the recently explored innovations used against resilient bacteria. The new approaches were based on the use of lipid polymer nanoparticles, nonlamellar liquid crystalline nanoparticles, oligonucleotides, natural compounds combination, smart materials, cationic peptides, antimicrobial photodynamic therapy, combined antibiotics therapy, nano-antibiotics and phage therapy. However, despite the extensive research done and the promising results of using nanosystems as drug delivery agents in the therapeutic management of infections, very limited formulations have been proposed for clinical trials. Thus, we recommend further steps to be taken in the way of putting such nanosystems formulations on the market.

## Figures and Tables

**Table 1:** Examples on recently published studies on different types of liposomes used against bacterial biofilm infections.

Liposome Type	Lipid Composition	Anti-Bacterial Drug/Nutraceutical Agents	Size	Biofilm	Findings	Reference Year
Conventional Liposomes	DPPC (Dipalmitoylphosphatidylcholine)	Calcifediol (25 (OH)D)	151.2 ± 0.3 nm	*P. aeruginosa*	Enhanced protection of liposomal 25(OH)D against *P. aeruginosa* infection. Increasing effective solubility and stability of 25(OH)D after incorporation in liposome preparation. High stability of colloidal parameters pre- and post-nebulization. Effective targeting to the bronchial cells, where infection and inflammatory responses are mostly localized in CF patients. Higher bacterial killing property against Pseudomonas-infected human 16- HBE cells compared with both empty liposomes and 25(OH)D solution in ethanol.	2017 [37]
Conventional Liposomes	Egg yolk soybean L-α phosphatidyl choline	Ampicillin Ofloxacin	280 nm–1.76 µm	Ocular post-surgery infections	Supercritical assisted liposome formation technique improved EE% (97%–99%) and used for production of more stable liposomes (up to 3 months). Further developments (in vitro/in vivo) will be needed to evaluate the anti-microbial effect of the proposed formulation.	2018 [78]
Conventional Liposomes	pH-sensitive lipids (PSLs):Phosphatidylcholine (PCS 100):Cholesterol (1:3:1 w/w/w)	Vancomycin	99.38 ± 0.59–105.60 ± 5.38 nm	MRSA	Successful synthesis of new biocompatible pH-sensitive lipids (PSLs). Improved targeted liposomal delivery of an antibiotic at the infection site. High encapsulation efficiency and loading capacity (29%–45% and 2.8%–4.5%, respectively). Structural changes in lipids at acidic pH caused the deformation of liposome structure and subsequent fast antibiotic release. Enhanced in vitro antimicrobial activities (low MIC values at pH 6.5). Better in vivo antibacterial activity (log10 cell forming unit CFU/mL of MRSA recovered from liposomal formulation in treated mice were 1.5- and 1.8-fold lower than that found in bare antibiotic treated ones.	2018 [79]
Conventional liposomes Deformable liposomes. Propylene glycol liposomes. Cationic liposomes.	Lipoid Lipoid + SDch Lipoid + PG DPPCT (Dipalmitoylphosphatidylcholine), DODAB (Dimethyldioctadecylammonium bromide)	Azithromycin	132–217 nm	MRSA	Major effect of the composition of phospholipid and presence of surfactant or propylene glycol on the physical characteristics of the liposomes, the in vitro drug release profile, deposition inside the skin, as well as in vitro antibacterial efficacy. Better drug retaining of liposomal formulations inside the skin compared to control. Good biocompatibility of liposomal formulations with keratinocytes and fibroblasts. Efficient MRSA inhibition of liposomes, 32 folds lower MIC, superior to bare drug.	2018 [35]
Conventional Liposomes	Hydrogenated soy phosphatidylcholine and cholesterol (7:3 w/w)	Ciprofloxacin nanocrystals	∼130 nm	*P. aeruginosa*.	High encapsulation efficiency of the drug nanocrystals (71%–79%). Prolonged drug release from the liposomes. Delivering this formulation in the osmohaler lead to exceed the MIC (10 folds) over a 24-h period.	2019 [80]
Conventional Liposomes	P90 G (Phospholipon 90 G), cholesterol	Salvia triloba. Rosmarinus officinalis.	∼200 nm	*K. pneumoniae*	Better enhancing of biopharmaceutical properties of essential oils by decreasing their volatility and improving their stability. High antibacterial activity compared to the unformulated essential oils.	2019 [81]
Conventional Liposomes	Phospholipon 90 G	Biosurfactants isolated from Lactobacillus gasseri Bc9	<200 nm	MRSA	Higher ability to eradicate *S. aureus* biofilm compared to free biosurfactant. Improved potential for local prevention of cutaneous infections.	2019 [82]
Conventional Liposomes	Egg yolk Lecithin	Cinnamaldehyde	75–92.14 nm	*S. aureus*	Long-term antibacterial activity and enhanced stability of cinnamaldehyde-loaded liposomes compared to unformulated one.	2019 [83]
Fusogenic Liposomes	Dope/Dppc/CHe MS (4:2:4 molar ratios)	Fusidic acid	98.77–99 nm	*S. epidermidis* (*Staphylococcus epidermidis*), *Acinetobacter baumannii*	Enhanced cell membrane permeability. Better targeting ability of fusidic acid to infection sites. Superior antibacterial activity of liposomal preparation against both Gram-positive and Gram-negative strains compared to free fusidic acid that was active only against Gram-positive strains, The lowest MICs were obtained against *S. epidermidis* (≤0.15 µg/mL) and *Acinetobacter baumannii* (37.5 µg/mL).	2015 [84]
Surface-Modified Liposomes	PC: DSPE PEG: Chol: SA (6.5:0.5:2:1 mole %)	β-Lapachone	88.7–112.4 nm	MRSA *C. neoformans (Cryptococcus neoformans*)	High drug encapsulation efficiency (97.4%–98.9%). Liposomal formulation did not interfere with drug antibacterial activity in addition to better improvement in its antifungal properties. Further in vivo studies should be done.	2015 [85]
Conventional liposomes Surface-Modified Liposomes Immobilized at surface of chitosan nanofiber mesh	Dipalmitoylphosphatidylcholine (DPPC)- cholesterol DPPC, cholesterol, DSPE-PEG-Mal and PE-Rho). Mal; (maleimide), Rho; (Rhamnolipids)	Gentamicin	126.25–140.26 nm	*E. coli* *P. aeruginosa* *S. aureus*	Good encapsulation efficiency (17%). Sustained drug release over 16 h. Better protection of drug from degradation. Decreased risk of toxicity. Promising performance for wound dressing applications.	2015 [86]
Surface- Modified Liposomes	Phospholipid, cholesterol, tween 80, vitamin E (6:1:1.8:0.12 mass ratios).	Gallic acid	153.2 ± 1.4 nm	*E. Coli* *S. aureus*	More favorable storage stability and higher antibacterial activity of lactoferrin gallic acid liposomes compared with Gallic acid liposomes. Improved the potential of lactoferrin liposomes as an effective delivery system for nutraceuticals in foods.	2019 [87]
Surface- Modified Liposomes	Phospholipids + Rhamnolipids (1:0, 10:1, 5:1, 2:1, 1:1 w/w).	Curcumin	46.4–251 nm	-	High loading efficiency (>90%) and loading capacity (3.5%). Good thermal and photochemical stability of curcumin after incorporation within liposomes. Prolonged sustained release of curcumin when rhamnolipids were incorporated. Further in vitro/in vivo studies against clinical strains are required.	2019 [88]
Surface-Modified Liposomes	DPPC/CH/PG/PE (8:10:1:2 mol/mol) Wheat germ agglutinin cyclodextrins	Ciprofloxacin Betamethasone	∼100 nm	*Aggregatibacter actinomycetemcomitans*	High encapsulation of both hydrophobic and hydrophilic drugs achieved. Better attachment to oral cells and controlled co-drug release in saliva. Synergistic therapeutic activity in bacteria-infected oral cells up to 24 h.	2020 [44]
Liposomes-in-Hydrogel	Phospholipids (PC)	Isoniazide N-Dodecanoyl isonicotinohydrazide (DINH)	∼130 nm	*M. tuberculosis*	Successful formulation of a thermo-responsive and self-healing liposome-in-hydrogel system for localized treatment of bone TB. Prolonged in vitro/in vivo drug release behavior. High biocompatibility for in vivo applications.	2019 [89]
Reactive Liposomes Encapsulating Enzyme (s)	DPPC, cholesterol, hexadecylamine	Endolysins	303 nm	Salmonella Typhimurium E. coli	High encapsulation efficiency of 22.81%–35.27%. Cationic liposomal preparation enhanced anti-bacterial activity of endolysins against Gm (-ve) organisms.	2019 [63]

**Table 2:** Examples on recent studies on lipid-based nanoparticles against bacterial infections.

Lipid Composition	Drug	Size	Biofilm	Findings	Reference Year
**SLNs**					
Compritol 888 ATO (a lipid excipient)	Vancomycin- Linoleic acid complex	102.7 ± 1.01	*S. aureus* & MRSA	Significant enhancement of drug encapsulation efficiency upon ion pairing with linoleic acid compared to free drug (70.73 ± 5.96% and 16.81 ± 3.64%), respectively. Superior antibacterial activity of complex loaded SLNs compared to free drug loaded SLNs against *S. aureus* (MIC = 31.25 and 250 µg/mL, respectively). Superior antibacterial activity of complex loaded SLNs against MRSA (MIC= 15.62 µg/mL).	2014 [101]
Compritol 888 ATO	Clotrimazole- silver complex	124.1 ± 2.5 nm	*S. aureus* & MRSA	Controlled drug release in both complex and free drug loaded NPs, with slower release for free drug loaded NPs (22% compared to 97% after 72 h). Biosafety of the synthesized clotrimazole silver complex to mammalian cells (cell viability >80%). Superior antibacterial activity of clotrimazole complex compared to free drug (MIC = 9.76 and 31.25 µg/mL, respectively) against *S. aureus*, and 15.62 and 31.25 µg/mL against MRSA, respectively. Clotrimazole SLNs completely lost its antibacterial activity after 36 h. Clotrimazole-silver SLNs had an MIC value of 52 µg/mL up to 54 h.	2015 [102]
Glyceryl monostearate Precirol Stearylamine	Rifampin	101 ± 4.7 nm	*S. epidermidis*	High encapsulation efficiency (about 70%). SLN formulations were more effective in biomass reduction compared to the free form	2016 [103]
Stearic acid	Levofloxacin	237.82 nm	*S. aureus* *E. coli*	Box–Behnken design applied to optimize the formulation. High entrapment efficiency (78.71%). Enhanced flux across excised goat cornea. Biphasic pattern of drug release. Formulation of non-irritant and safe NPs for topical ophthalmic use. Comparable antibacterial activity against *S. aureus* and *E. coli* relative to marketed eye drops. In vivo studies for levofloxacin-SLN should be carried out to determine its potential for ophthalmic delivery.	2016 [104]
Glyceryl behenate Tripalmitin Stearic acid	Clarithromycin	318–526 nm.	*S. aureus*	High drug content in a range of 63%–89%. Burst drug release followed by extended drug release up to 48 h. The carbon chain length of lipid has a great impact on particle size, drug content and release rates of SLNs. SLN formulations rendered clarithromycin, 2 times more effective against its target (microdilution method). Antibacterial activity confirmed by comparative zones of inhibition around SLN (Solid lipid nanoparticles) formulations wells with clarithromycin zone.	2019 [90]
Glycerol monostearate	Furosemide-silver complex (Ag-FSE)	129.8 ± 38.5 nm	*P. aeruginosa* *S. aureus*	High encapsulation efficiency (∼93%) of complex loaded SLNs. Sustained drug release over 96 h. Improvement of antibacterial activity of complex loaded SLNs (2 and 4 folds) against *P. aeruginosa* and *S. aureus*, respectively.	2019 [105]
**NLCs**					
pH responsive NLC (Stearic acid and oleic acid)	Vancomycin	225.2 ± 9.1 nm	*S. aureus* (Resistant and Sensitive)	Improved antibacterial activity of drug loaded-NLCs against *S. aureus* and MRSA than the free drug. Better killing percentage of NLcs (2.5-fold) higher than the bare drug. High bactericidal activity in vivo (mouse model of MRSA skin infection) with 37-fold reduction in MRSA CFU (colony-forming unit) load of the skin treated with nanostructured lipid carriers (NLCs) compared to free vancomycin.	2019 [106]
Cetyl palmitate and caprylic acid	Mupirocin	99.8–235 nm	MRSA	Improved antimicrobial activity against *Streptococcus pyogenes* and *S. aureus* compared to free drug. No signs of toxicity on albino rats. Improved pharmacokinetic parameters due to protection of the drug against enzymatic degradation.	2019 [107]
Stearic acid and oleic acid NLC with unmodified surface. NLC surface functionalized with a tuftsin-modifed peptide	Rifampicin	NLC: 210 ± 8 nm Surface modified NLC: 285 ± 11 nm	*M. tuberculosis*	Significant internalization by macrophages obtained with surface modified NLc. Both surface-modified or unmodified NLC were 2-fold more effective against *M. tuberculosis (Mycobacterium tuberculosis)* than free rifampicin.	2019 [108]

**Table 3:** Examples on recent studies on polymeric micelles against bacterial infections.

Composition	Drug	Size	Biofilm	Findings	Reference Year
Cholesterol conjugated Poly (ethylene glycol) and anchored with transcriptional activator TAT (a trans-activator of transcription) peptide	Ciprofloxacin	180 nm	Streptococcus pneumoniae E. coli Neisseria meningitides	Better uptake of micelles by human astrocytes due to the presence of TAT. Enhanced ability of micelles to cross the BBB and enter the brain for treatment of brain infections.	2008 [114]
Poly (lactic acid-co-glycolic acid)-block-poly (ethylene glycol)-alendronate copolymer	Vancomycin	39.62–55.08 nm	*S. aureus*	The conjugation of alendronate to the micelle surface did not affect drug loading capacity neither it’s in vitro release behaviors. Appropriate cytotoxicity. Enhanced bone targeted delivery of vancomycin to treat osteomyelitis.	2015 [115]
Mixed-shell-polymeric-micelles consisting of a hydrophilic PEG-shell and pH-responsive poly (β-amino ester)	Triclosan	160 nm	*S. aureus*	Increased ability to target *S. aureus* biofilm owing to their stealth properties at physiological pH. Presence of pH responsive moiety makes the particles acquire a positive charge under low pathological pH conditions facilitating binding with bacterial cell surfaces. Release of encapsulated drug occurs as a result of hydrolysis by bacterial lipases.	2016 [116]
Silver decorated amphiphilic diblock copolymers, poly (ε-caprolactone)-block-poly(aspartic acid)	Curcumin	90–95 nm	*P. aeruginosa S. aureus*	Synergistic antibacterial activities against both Gram-negative and Gram-positive bacterial strains on contrary to either sliver micelle or curcumin-loaded micelle alone. Slow drug release rate in the absence of lipase, compared to about 95% release over 48 h when incubated with *P. lipase* High biocompatibility with RBCs.	2017 [117]
Ethylene oxide-propylene oxide triblock copolymers, Pluronics^®^ (P84, P85, P103, P105, P123 and F127)	Rifampicin and Isoniazid	-	*M. tuberculosis*	Controlled drugs release. Improved antibacterial activity of drugs loaded micelles against *M. tuberculosis* relative to corresponding free drug solutions. Enhanced drug permeability from micelles across Caco-2 monolayer compared to bare drugs.	2018 [118]
Amphiphilic poly (ethylene glycol)-poly(*ε*-caprolactone) copolymers conjugated with vancomycin.	Ciprofloxacin	77 nm	*P. aeruginosa*	Enhanced blood circulation and bacterial targeting due to PEG shell and vancomycin. Improved release of encapsulated antibiotic at the infection site as a result of hydrolysis by bacterial lipase. High bactericidal activity in vivo (*P. aeruginosa*-infected mice) compared with free drug and micelles without vancomycin moiety. Three doses of drug loaded micelles almost restored the normal alveolar microstructure.	2018 [119]
Fatty acid grafted chitosan conjugates nanomicelles	Ciprofloxacin	260 nm	*P. aeroginosa* *K. pneumoniae* *S. pneumoniae*	High drug loading (about 19%). Enhanced MIC (4 and 2 times) was lower than the free drug against *P. aeroginosa* and *K. pneumoniae*, respectively.	2018 [120]
Carboxy methyl chitosan hydrophobically modified with stearic acid and conjugated with urea	Clarithromycin	200 nm	*H. pylori*	Better targeting to H. pylori due to the grafted ureido groups. High drug encapsulation efficiency (85.83 ± 0.98%). Prolonged drug release. In vitro inhibitory assay indicated a significant enhancement in anti-*H. pylori* activity.	2019 [121]
D-α-tocopherol polyethylene glycol 1000 succinate polymeric micelles	Baicalin	14.05 ± 4.52 nm,	*E. coli*	In vivo study suggested the potential of baicalin loaded polymeric micelles to suppress the periodontal damage and alveolar bone loss compared to free drug.	2019 [122]

**Table 4:** Examples on recent studies on polymeric nanoparticles against bacterial infections.

Type of Polymeric Nanoparticles	Drug	Size	Biofilm	Findings	Reference Year
**Natural polymers**					
Chitosan	Ciprofloxacin Chlortetracycline HCL. Gentamycin sulfate.		*S. aureus* *E. coli*	Enhanced antibacterial activity of chitosan nanoparticles (NPs) loaded with antibiotics. Higher antibacterial activity against Gram-positive bacteria than Gram-negative bacteria. Order of inhibition is: Gentamycin sulfate ˃ ciprofloxacin HCL ˃ Chlortetracycline hydrochloride	2015 [131]
pH-responsive chitosan coated iron oxide NPs	Ciprofloxacin	30–80 nm	urinary tract and intestinal infections	High drug loading efficiency (99%). Sustained drug release over 5 days. In vitro/in vivo antibacterial activity need to be evaluated.	2016 [132]
Genipin cross-linked chitosan/heparin NPs	Ciprofloxacin	250 nm	*E. coli* MTCC 443	Improved drug loading efficiency (35.5 ± 2.5 to 45.5 ± 3.0%). Genipin crosslinking sustained drug release in acidic pH (16% after 2 h). Enhanced antibacterial activity compared to free drug (MIC equals 0.125 and 0.25 mg/mL, respectively). In vivo study needs to be evaluated.	2016 [133]
Chitosan/fucoidan NPs	Gentamicin	270–300 nm	*K. pneumoniae*	High drug encapsulation efficiency (91%–94%). Superior enhancement of antibacterial activity MIC of drug-loaded NPs was 1.95 µg/mL compared to >62.5 µg/mL for free drug. Enhanced bioavailability 1.8-fold increase in drug C_max_ after its encapsulation in NPs.	2016 [134]
pH-responsive chitosan nanoparticles with new anionic gemini surfactant (AGS)	Vancomycin	220.57 ± 5.9 nm	MRSA	Improved encapsulation efficiency percent (59.89 ± 2.33%). Sustained drug release at acidic and normal physiological conditions. Enhanced antibacterial activity in vitro at pH 6.5 than pH 7.4 (MIC values, 7.81 and 62.5 µg/mL respectively). High in vivo anti MRSA activity (mice skin infected model) than free vancomycin (8 folds).	2017 [135]
Chitosan nanoparticles and fucoidan coated chitosan NPs	Ciprofloxacin	Chitosan NPs: 124 ± 7 nm. Fucoidan coated chitosan CNPs: 320 ± 18 nm.	Salmonella	Low encapsulation efficiency and loading efficiency of the prepared NPs (10.6 ± 0.6% and 5.2 ± 0.4%, respectively). Sustained drug release over 2 weeks. Coating with fucoidan enhanced drug delivery within macrophages. Superior antibacterial activity of fucoidan coated NPs in vivo (*Salmonella Paratyphi,* an infected Drosophila melanogaster fly model), the microbial load decreased by 95% more than the free drug.	2017 [136]
Chitosan-Dextran sulphate NPs	Ciprofloxacin	350 nm	Gm +ve and Gm -ve ophthalmic microorganisms.	High drug encapsulation efficiency (83%/wt). Monotonous controlled release for 21 h. Powerful antibacterial activity of Cipro-NPs than the bare ciprofloxacin. Ocular irritancy test revealed that the prepared nanoparticles were non-irritant.	2017 [137]
Alginate lyase functionalized chitosan NPs	Ciprofloxacin	205.5 ± 9.0 nm	*P. aeruginosa*	Improved encapsulation efficiency percent (51.8 ± 2.1%). Sustained drug release. Prolonged MIC and MBEC (minimal biofilm eradication concentration) (0.125 µg/mL and 0.5 µg/mL after 24 h), respectively. Significant reduction in biofilm aggregation. Safe preparation on the lung of rats. Further in vivo assessment on infected animals is required.	2019 [138]
**Synthetic polymers**					
PLGA functionalized with DNase I	Ciprofloxacin	251.9 nm	*P. aeruginosa*	Controlled drug release Successful targeting and destroying of the biofilm by degrading the extracellular DNA. Successful reduction of biofilm mass, size and living cell density. Minimal cytotoxicity. Complete eradication (99.8%) of established biofilm upon repeated nanoparticle administration over three days. Considered as novel antimicrobial nanoparticles to treat persistent bacterial infections	2015 [139]
PLGA	Amikacin	447 ± 7	*P. aeruginosa*	High encapsulation efficiency percent (76.8 ± 3.8%). No toxicity against RAW macrophages until 24 h of exposure. Reduced antibacterial activity of NPs against planktonic cells (MIC:16 μg/mL and MBC: 32 μg/mL) compared to free drug (MIC: 4 μg/mL and MBC: 8 μg/mL), due to gradual drug release from NPs. Reduced Antibacterial activity of NPs against biofilm (MBEC: 512 μg/mL) versus the free amikacin (MBEC 128 μg/mL).	2016 [140]
PLGA	Ciprofloxacin-SDS complex (ciprofloxacin complex loaded PLGA)	190.4 ± 28.6 nm	*P. aeruginosa*	High encapsulation efficiency percent (79%). The complex-loaded NPs were non-toxic at concentrations >>MICcipro against bacterial strains. Enhanced antibacterial activity of the complex NPs relative to free drug (zone of inhibition 36.0 ± 0.8 and 32.0 ± 0.5 mm, respectively).	2017 [141]
PLGA (poly(lactic-co-glycolic acid)	Gentamicin	227 nm	*K. pneumonia*	Drug encapsulation efficiency (135 μg/mg PLGA). Reduced anti-microbial activity of drug loaded nanoparticles relative to free drug (MIC = 10.94 and 1.09 and μg/mL, respectively). Enhanced anti-microbial activity of drug loaded NPs were observed over 120 h incubation (MBC = 5.47 μg/mL). In vivo study using Galleria mellonella larvae model showed that the nanoparticle formulation was as effective as the free drug in vivo.	2018 [142]
PLGA and PEG-PLGA di-block NPs	Tobramycin	NPs: 225–231 nm MPs: 896–902 nm	*P. aeruginosa* and *Burkholderia cepacia complex (Bcc)*	Low encapsulation efficiency (about 3%). Powerful bactericidal activity compared to free drug (less than 0.77 mg/L encapsulated drug required to kill bacteria in the biofilm, whereas 1000 mg/L of free drug needed. No cytotoxicity was detected in vitro in human lung epithelial cells.	2018 [143]
Polyethylenimine/diazeniumdiolate (PEI/NONOate)-doped PLGA nanoparticles	Nitric oxide (NO)	240 ± 20	MRSA	The amount of drug loaded in NPs (122 ± 1 μmole/g NPs). Extended NO release over 4 days in simulated wound fluid. High MIC (0.625 mg/mL). High cell viability over 80% after NPs treatment indicating absence of toxicity to mammalian fibroblast cells (L929) compared to commercially available topical antiseptics. Superior wound healing activities in diabetic ICR (Institute of Cancer Research) mice and in Balb/c mice (an albino, laboratory-bred strain) (>90% wound closure 12 days’ post injury).	2019 [144]
Alginate modified-PLGA nanoparticles	Amikacin and moxifloxacin	Alginate coated PLGA NPs: 640 ± 32 nm Alginate entrapped PLGA NPs: 312–365 nm	*M. tuberculosis* (H37Ra)	Enhanced anti-mycobacterial activity of the dually entrapped drug-loaded particles (bacterial viability was 0.6%, compared to 6.49% for amikacin NPs and 3.27% for moxifloxacin NPs). Further in vivo evaluation should be done.	2019 [145]

**Table 5:** Nanosystem-based antibiotics and anti-toxins in clinical trials different stages.

Antibiotic	Clinical Trial	Medical Condition/Indication	Trial Phase	Intervention Treatment
Ciprofloxacin	Inhaled ciprofloxacin loaded-liposome: Once a day management of respiratory infections [54].	*P. aeruginosa*	Phase 1	Ciprofloxacin
Ciprofloxacin	Inhaled ciprofloxacin loaded-liposome: Once a day management of respiratory infections [54].	*P. aeruginosa*	Phase 2a	Ciprofloxacin
Ciprofloxacin	Inhaled ciprofloxacin loaded-liposome in patients with non-cystic fibrosis bronchiectasis and chronic lung infection with Pseudomonas aeruginosa (ORBIT-3 and ORBIT-4): two phase 3, randomised controlled trials [208].	Bronchiectasis and Chronic *P. Aeruginosa* Infection	Phase 3	Inhaled Liposomal Ciprofloxacin
Ciprofloxacin	Phase 3 Study with Ciprofloxacin Dispersion for Inhalation in Non-Cystic Fibrosis Bronchiectasis (ORBIT-3) [209].	Non-Cystic Fibrosis Bronchiectasis	Phase 3	Ciprofloxacin dispersion for inhalation (Liquid mixture of liposomally encapsulated and un encapsulated ciprofloxacin) Placebo: Liquid formulation of empty liposomes.
Amikacin	Liposomal Amikacin for Inhalation (LAI) for Nontuberculous Mycobacteria [210].	Mycobacterium Infections, Nontuberculous	Phase 2	Liposomal amikacin for inhalation (LAI) Drug: placebo
Amikacin	Extension Study of Liposomal Amikacin for Inhalation in Cystic Fibrosis (CF) Patients with Chronic Pseudomonas Aeruginosa (Pa) Infection [213].	Cystic Fibrosis Patients with Chronic *Pseudomonas aeruginosa* Infection	Phase 3	Amikacin
Amikacin	Inhaled amikacin loaded-liposome for treating Mycobacterium Abscesses Lung Disease [212].	Mycobacterium Infections, Nontuberculous Mycobacteria, Atypical	Phase 2	Liposomal amikacin for inhalation (LAI) plus multi-drug regimen
Amikacin	Study to Evaluate Efficacy of inhaled amikacin loaded-liposome combined with multi-drug regimen, Compared to Multi-drug Regimen Alone (CONVERT) [218].	Mycobacterium Infections, Nontuberculous	Phase 3	Liposomal Amikacin for Inhalation, 590 mg
Amikacin	Study of Dose Escalation of Liposomal Amikacin for Inhalation (ARIKAYCE™)-Extension Phase [211].	Cystic Fibrosis	Phase 2	Drug: Arikayce™
Biological: CAL02	CAL02; a liposomal adjunctive anti-toxin therapy in infections. A new therapeutic approach for severe community-acquired pneumonia [216].	Severe community-acquired pneumonia	Phase 2 and 3	CAL02 anti-toxin
Biological: GS-CDA1 Biological: MDX-1388	Study of the Clinical Effectiveness of a Human Monoclonal Antibody to C. Difficile Toxin A and Toxin B in Patients with Clostridium Difficile Associated Disease [219].	*Clostridium Difficile* Associated Disease	Phase 2	Biological: (GS-CDA1) Biological: MDX-1388

**Table 6:** Nanosystem-based antimicrobial peptides in clinical trials different stages.

Antimicrobial Peptides	Medical Condition/Indication	Clinical Trial Phase	Antimicrobial Peptides Source
Mutacin 1140 (MU1140) [220]	Gm +ve bacteria (MRSA, *C. difficile (Clostridium difficile*))	Preclinical	*Streptococcus mutans*
lipohexapeptides 1345 (HB1345) [217]	Broad-spectrum antibiotic, acne	Preclinical	Lipopeptide
Novarifyn (NP432) [50]	MRSA, *P. aeruginosa, C. difficile, A. baumannii, E. coli*	Preclinical	Synthetic antimicrobial
Arenicin (AP139) [221]	Gm−ve bacteria, UTI	Preclinical	Lugworm Arenicol marina
Arenicin (AP138) [217]	MRSA implant infections	Preclinical	Arenicin analog
Arenicin (AP114) [217]	*C. difficile*	Preclinical	Arenicin analog
Avidocin and purocin [222]	Gm+ve and Gm−ve bacteria	Preclinical	Modified R-type bacteriocin
Novacta biosystems (NVB-302) [223]	*C. difficile*	Phase 1	Lantibiotic
Human lactoferrin (hlf1-11) [224]	Infection following transplantation	Phase 1 and phase 2	Lactoferricin analog
(a potent cyclic lipodepsipeptides antibiotic) Wap-8294A2 [225]	Gm+ve bacteria (VRE and MRSA)	Phase 1 and phase 2	Lysobactor spp.
The specifically targeted antimicrobial peptide (C16G2) [226]	Prevention of tooth decay caused by Streptococcus mutans	Phase 2	Synthetic peptide
Antimicrobial Peptide (DPK-060) [227]	Acute external otitis	Phase 2	Human protein kininogen
LTX-109 (Lytixar) [228,229]	Nasal decolonization of MRSA Impetigo	Phase 1 & 2 Phase 2	Synthetic peptidomimetic
p2TA (AB 103) [230] (A CD28 mimetic peptide)	Necrotizing soft tissue infections	Phase 3	Synthetic peptide
Surotomycin [231]	*C. difficile* (diarrhea)	Phase 3	Cyclic lipopeptide
Ramoplanin (NTI-851) [232]	*C. difficile*	Phase 2	Actinoplanes spp
